# Draft Genome Sequence of *Ardenticatena maritima* 110S, a Thermophilic Nitrate- and Iron-Reducing Member of the *Chloroflexi* Class *Ardenticatenia*

**DOI:** 10.1128/genomeA.01347-15

**Published:** 2015-11-19

**Authors:** James Hemp, Lewis M. Ward, Laura A. Pace, Woodward W. Fischer

**Affiliations:** a Division of Geological and Planetary Sciences, California Institute of Technology, Pasadena, California, USA; b Department of Medicine, University of California San Diego, La Jolla, California, USA

## Abstract

We report here the draft genome sequence of *Ardenticatena maritima* 110S, the first sequenced member of class *Ardenticatenia* of the phylum *Chloroflexi*. This thermophilic organism is capable of a range of physiologies, including aerobic respiration and iron reduction. It also encodes a complete denitrification pathway with a novel nitric oxide reductase.

## GENOME ANNOUNCEMENT

*Ardenticatena maritima* 110S was originally isolated from an iron-rich coastal hydrothermal field in the Kirishima Volcanic Belt of Japan (1). Closely related strains have been reported from hot springs (2) and hydrothermal vents (3). *A. maritima* is a filamentous nonmotile organism that can facultatively reduce nitrate and iron (1). It grows optimally at 50 to 70°C and pH 7.0 (pH range, 5.5 to 8.0).

The genome of *A. maritima* 110S (DSM 23922) was sequenced as part of a project to expand the phylogenetic breadth of *Chloroflexi* genomes. Genome sequencing was performed at SeqMatic using the Illumina MiSeq sequencing platform. SPAdes 3.1.1 (4) was used to assemble the genome. The genome was screened for contaminants based on sequence coverage, G+C composition, and BLAST hits of conserved single-copy genes. Genome annotation was performed using the NCBI Prokaryotic Genome Annotation Pipeline. The draft genome is 3.62 Mb in size, assembled into 12 contigs. It contains 3,041 genes, 2,578 coding sequences (CDSs), 2 16S RNAs, 46 tRNAs, and 10 clustered regularly interspaced short palindromic repeat (CRISPR) arrays. It is estimated to be ~99% (111/111) complete based on conserved single-copy genes.

Analysis of the *A. maritima* genome revealed the presence of many genes responsible for its physiological breadth*. A. maritima* encodes a branched aerobic respiratory chain, including complex I (NADH dehydrogenase), complex II (succinate dehydrogenase), complex III (cytochrome *bc* complex), and three oxygen reductases (A- and B-family heme-copper oxygen reductases and *bd* oxidase). B-family heme-copper oxygen reductases are commonly found in aerobic thermophiles, enabling growth with the low oxygen levels found in thermal systems (5). *A. maritima* also encodes a complete denitrification pathway composed of nitrate reductase (NapA), nitrite reductase (NirK), a novel nitric oxide reductase (eNOR) (6), and nitrous oxide reductase (NosZ). Interestingly, the eNOR is found in an operon with NirK, suggesting that these genes are coregulated. No genes were found for either lipopolysaccharide (LPS) biosynthesis or outer membrane proteins, consistent with the proposal that *Chloroflexi* have only one membrane (7).

### Nucleotide sequence accession number.

This whole-genome shotgun project has been deposited in DDBJ/EMBL/GenBank under the accession no. LGKN00000000.
